# Current News

**Published:** 1899-12

**Authors:** 


### Current News.

### INTERNATIONAL DENTAL CONGRESS.

THE Transportation Committee of the National Dental Association for the International Dental Congress in Paris, next year, are perfecting arrangements for tours and special rates for delegates and their families, and in all probability they will be completed so as to appear in the January issue of the journals.

W. E. GRISWOLD,
*Secretary.*

DENVER, COL.

---

### OHIO STATE DENTAL SOCIETY.

THE Thirty-fourth Annual Meeting of the Ohio State Dental Society will be held at the Grand Southern Hotel, Columbus, Ohio, December 5, 6, and 7, 1899. A good programme, consisting of essays and clinics, has been prepared. A cordial invitation is extended to the profession at large.

HENRY BARNES,
*Chairman Executive Committee.*

### NATIONAL SCHOOL OF DENTAL TECHNICS.

THE meeting of the National School of Dental Technics will be held in Philadelphia, at the Continental Hotel, beginning at ten A.M., Wednesday, December 27, and continuing three days.

Every teacher in the profession should be present. A most excellent programme will be presented, consisting of a lecture and demonstration by Professor J. Liberty Tadd, and papers by Drs. Faneuil D. Weisse, C. S. Case, D. A. Gritman, A. E. Webster, W. H. Whitslar, M. H. Cryer, H. J. Goslee, Otto Arnold, I. N. Broomell, G. V. Black, A. H. Thompson, James Truman, and others.

GEO. H. WILSON.

---

### MARYLAND STATE DENTAL ASSOCIATION.

THE following are officers of the Maryland State Dental Association for 1899–1900:

President, E. E. Cruzen, D.D.S.; First Vice-President, G. Marshall Smith, D.D.S.; Second Vice-President, J. K. Burgess, D.D.S.; Recording Secretary, Richard Grady, M.D., D.D.S.; Corresponding Secretary, George R. Carter, D.D.S.; Treasurer, S. C. Pennington.

*Executive Committee.*—B. Holly Smith, M.D., D.D.S., Chairman, C. M. Gingrich, D.D.S., and W. W. Dunbracco, D.D.S.

RICHARD GRADY,
*Recording Secretary.*

720 N. HOWARD STREET, BALTIMORE, MD.

---

### MISSOURI STATE DENTAL ASSOCIATION.

THE Missouri State Dental Association, at its Thirty-fifth Annual Meeting, Kansas City, July 11–14, 1899, elected the following officers for the coming year:

President, Dr. W. L. Reed, Mexico; First Vice-President, Dr. S. J. Smith, Columbia; Second Vice-President, Dr. A. M. Tutt, Liberty; Corresponding Secretary, Dr. B. L. Thorpe, St. Louis; Recording Secretary, Dr. H. H. Sullivan, Kansas City; Treasurer, Dr. J. A. Price, Savannah.

The next meeting will be held at Louisiana, Mo., on the first Tuesday after July 4, 1900.

B. L. THORPE,
*Corresponding Secretary.*

